# Supplementary material for: Genome-Wide Mining of CULLIN E3 Ubiquitin Ligase Genes from Uncaria rhynchophylla
Source: Plants (Basel). 2024 Feb 15;13(4):532. doi: 10.3390/plants13040532 (PMC10891735; doi:10.3390/plants13040532)
Supplement: Supplementary file 1 [file plants-13-00532-s001.zip › Table S1. The detailed information of 12 UrCUL gene.pdf]

Table S1. The Detailed information of 12 *UrCUL* genes in *U. rhynchophylla* genome

Gene ID	Gene Name	Chr	Start	Stop	Number of Amino Acid	Molecular weight	Theoretical pI	Instability index	Subcelluar Localization
g35191.t1	UrCUL1	chr_7	921815	928686	739	85893.7	6.71	33.87	Nucleus
g9477.t1	UrCUL1-likeA	chr_8	861797	869123	710	82572.71	6.15	40.01	Nucleus
g38913.t1	UrCUL1-likeB	chr_19	12614040	12628826	1367	158900.64	5.77	41.33	Nucleus
g17654.t1	UrCUL1-likeC	chr_22	15824354	15829246	548	64184.41	6.1	40.48	Nucleus
g17653.t1	UrCUL1-likeD	chr_22	15841304	15848397	742	86278.09	6.79	39.54	Nucleus
g17656.t1	UrCUL2-likeA	chr_22	15809890	15817740	709	82962.37	7.56	34.57	Nucleus
g17655.t1	UrCUL2-likeB	chr_22	15823519	15824315	173	20179.51	9.18	31.69	Nucleus
g2433.t1	UrCUL3A	chr_3	21972739	21976800	736	85405.78	7.01	47.83	Cytoplasm
g15448.t1	UrCUL3B	chr_10	2622338	2630359	1100	125120.69	6.27	49.47	Nucleus
g6507.t1	UrCUL4-likeA	chr_17	12547447	12570778	810	92974.77	6.69	42.45	Cytoplasm
g6004.t1	UrCUL4-likeB	chr_18	16272073	16311831	849	96860.16	7.57	43.49	Cytoplasm
g2463.t1	UrAPC2	chr_3	22208848	22217388	886	99766.11	4.76	44.68	Nucleus
