# Supplementary material for: Genome-Wide Mining of CULLIN E3 Ubiquitin Ligase Genes from Uncaria rhynchophylla
Source: Plants (Basel). 2024 Feb 15;13(4):532. doi: 10.3390/plants13040532 (PMC10891735; doi:10.3390/plants13040532)
Supplement: Supplementary file 1 [file plants-13-00532-s001.zip › Table S2. The syntenic analysis of UrCUL genes betwe.pdf]

Table S2. The syntenic analysis of *CUL* genes between *U. rhynchophylla* and *A.thaliana*, *O.sativa* and *C. camephora*

Gene_ID	Ur_Chrom	Gene_ID	Chrom	Species
g2433.t1	chr3	AT1G26830.1.TAIR10	Chr1	*Arabidopsis thaliana*
g2433.t1	chr3	AT1G69670.1.TAIR10	Chr1	*Arabidopsis thaliana*
g35191.t1	chr7	AT1G02980.1.TAIR10	Chr1	*Arabidopsis thaliana*
g35191.t1	chr7	AT4G02570.1.TAIR10	Chr4	*Arabidopsis thaliana*
g9477.t1	chr8	AT1G02980.1.TAIR10	Chr1	*Arabidopsis thaliana*
g9477.t1	chr8	AT4G02570.1.TAIR10	Chr4	*Arabidopsis thaliana*
g15448.t1	chr10	AT1G26830.1.TAIR10	Chr1	*Arabidopsis thaliana*
g15448.t1	chr10	AT1G69670.1.TAIR10	Chr1	*Arabidopsis thaliana*
g15448.t1	chr10	AT1G14300.2.TAIR10	Chr1	*Arabidopsis thaliana*
g6507.t1	chr17	AT5G46210.1.TAIR10	Chr5	*Arabidopsis thaliana*
g2433.t1	chr3	LOC_Os02g51180.1.MSUv7.0	Chr2	*Oryza sativa*
g2463.t1	chr3	transcript:CDP00362	Chr11	*Coffee canephora*
g2433.t1	chr3	transcript:CDP00322	Chr11	*Coffee canephora*
g35191.t1	chr7	transcript:CDP03526	Chr1	*Coffee canephora*
g35191.t1	chr7	transcript:CDO98884	Chr3	*Coffee canephora*
g9477.t1	chr8	transcript:CDP03526	Chr1	*Coffee canephora*
g9477.t1	chr8	transcript:CDO98884	Chr3	*Coffee canephora*
g15448.t1	chr10	transcript:CDP00323	Chr11	*Coffee canephora*
g17656.t1	chr22	transcript:CDP03526	Chr1	*Coffee canephora*
