# Supplementary material for: Genome-Wide Mining of CULLIN E3 Ubiquitin Ligase Genes from Uncaria rhynchophylla
Source: Plants (Basel). 2024 Feb 15;13(4):532. doi: 10.3390/plants13040532 (PMC10891735; doi:10.3390/plants13040532)
Supplement: Supplementary file 1 [file plants-13-00532-s001.zip › Table S3. The primers used for expression analysis.pdf]

Table S3. The primers used for expression analysis for 12 *UrCULs*

Gene_name	5'primer	3'primer
UrCUL1	ACGCAGAAGTATCCTAATGA	GAAGTGACCTCCGAATGA
UrCUL1-likeA	AACAGTCTTGCCATCTCTAA	CCACCTGACCATAACCTT
UrCUL1-likeB	CAACAAGAAGGCAGATAAGAG	CATTCACATAGGCGAGGTA
UrCUL1-likeC	TGATGATGTTGTGAGACTTC	TCTTCTTGATTGCCTTGAC
UrCUL1-likeD	TTGACAAGGATAGGCGATAT	CCATTACCAACTGCTGATAG
UrCUL2-likeA	AATGTGGTGGTCAGTTCA	CAGCGGTAAGGACAGTAA
UrCUL2-likeB	GATAGGCGATATGCTATTGATG	TCCATTACCAACTGCTGAT
UrCUL3A	ATTCAGGCTTGGTGGATAT	TTCTCGGCATCAGTAACA
UrCUL3B	CAAGTAGGCTATGAAGATGAAG	CTATGCTGCGTCTGTAGA
UrCUL4-likeA	AGGCTGTTGCTTACTCTC	AGGCTGTTGCTTACTCTC
UrCUL4-likeB	CACTCCTTCACCATCTTCT	TAACACCTTCAGCAGCATA
UrAPC2	ATGGTGTCTTCTGTTCTGA	ATTCGTCCAACCTTCCTT
